# Effects of Palliative Care Education on Healthcare Professional Students: An Umbrella Review

**DOI:** 10.1007/s40670-026-02640-8

**Published:** 2026-01-17

**Authors:** Lin Xiao, Zhishan Xie, Man Yin, Jinfeng Ding

**Affiliations:** 1 School of Nursing, Southern Medical University, Guangzhou, China; 2 JBI Evidence Based Nursing and Midwifery PR China, Guangzhou, China; 3 Faculty of Science, Medicine and Health, University of Wollongong, Northfields Ave, Wollongong, NSW 2500 Australia; 4 Xiangya School of Nursing, Central South University, 172 Tongzipo Road, Changsha, 410013 China

**Keywords:** Palliative care, Medical education, Overview, Kirkpatrick model

## Abstract

**Objective:**

To conduct an overarching review of previous reviews regarding the effects of palliative care education among healthcare students, with a focus on learning outcomes.

**Methods:**

Seven English databases and four Chinese databases were search for qualitative and quantitative systematic reviews from inception to May 2025. Two reviewers independently used the JBI Critical Appraisal Checklist for data extraction and appraisal. The effects of palliative care education were narratively synthesized against four levels of criteria according to the Kirkpatrick model.

**Results:**

Across all systematic reviews, the educational interventions predominantly focused on symptom management and communication skills, while limited attention was given to topics such as death and dying, ethical issues, spiritual and psychosocial care, legal considerations, and grief and bereavement. Positive outcomes related to students’ learning (Kirkpatrick level 2), including knowledge, attitudes, and skills, were reported. Additionally, four systematic reviews assessed students’ reactions to educational programs (Kirkpatrick level 1) and behavior changes (Kirkpatrick level 3). However, no systematic reviews explored the effects of the program on patient outcomes and organizational-level outcomes (Kirkpatrick level 4).

**Conclusion:**

Our review underscores the positive influence of palliative care education programs on the knowledge, attitudes, and skills of healthcare students. Furthermore, it highlights the significance of assessing the effectiveness of palliative care educational interventions on patients’ outcomes in the real-world.

**Supplementary Information:**

The online version contains supplementary material available at 10.1007/s40670-026-02640-8.

## Introduction

Palliative care represents an essential approach aimed at enhancing the quality of life for patients, including both adults and children, and their families grappling with the challenges posed by life-threatening illnesses [1]. This approach focuses on alleviating and preventing suffering by promptly identifying, assessing, and addressing physical, psychosocial, and spiritual pain and other associated issues [1]. The significance of palliative care is underscored by the 67th World Health Assembly (WHA) Resolution in 2024, which advocates for the integration of palliative care into all tiers of healthcare and the inclusion of such care in Universal Health Coverage plans across nations [2].

Regrettably, the global need for palliative care remains alarmingly unmet. An estimated 60 million individuals worldwide require palliative care annually, resulting in over 20 billion days of serious health-related suffering (SHS) [3]. Shockingly, only approximately 12% of those in need actually receive palliative care. Furthermore, this situation is poised to worsen, with the demand for palliative care expected to double by 2060 due to the global aging population and rising instances of cancer and other non-communicable diseases [4].

Meeting the rapidly increasing demand for palliative care through specialist providers alone appears to be an unlikely prospect. A promising solution involves equipping all healthcare providers with the fundamental skills, knowledge, and attitudes required for primary palliative care [5]. This approach entails training programs that encompass workplace-based continuing education and the integration of palliative care into the undergraduate and postgraduate student programs of health professional students. Remarkably, palliative care has often been overlooked or underemphasized in existing school curricula [6]. However, there is a growing acknowledgment of the need to fully integrate palliative care into educational and training frameworks to adequately prepare future healthcare professionals to meet clinical requirements [7].

Prior reviews have explored strategies for delivering palliative care training to health professional students and assessed their effectiveness [8–12]. Nevertheless, these reviews exhibit substantial methodological diversity and variability in synthesizing studies with differing designs and target populations. Notably, variations in reporting training effectiveness have also been observed among these reviews, which hampers our ability to form a comprehensive and coherent global understanding of the impact of palliative care training for health professional students. Consequently, it is imperative to leverage a standardized and validated framework to collate and organize outcome measures for effectiveness across these reviews.

To address these challenges and achieve our objectives, we aim to undertake an overarching review of previous reviews. This umbrella review aims to collate and appraise findings from existing systematic reviews on palliative care education for healthcare professional students, applying Kirkpatrick’s Four-Level Training Evaluation Model to categories educational outcomes. By mapping the current evidence to an established model widely used in medical and health professions education, this review provides educators and curriculum developers with strengthened guidance on effective strategies for integrating palliative care training into health professional students’ programs.

## Objective

We aimed to synthesize evidence from existing systematic reviews (SRs) on palliative care education for healthcare professional students by evaluating learning outcomes such as knowledge, skills, attitudes, and competence, and examining curriculum characteristics.

## Methods

A preliminary search of several databases (Joanna Briggs Institute Database of Systematic Reviews and Implementation Reports, the Cochrane Database of Systematic Reviews, PROSPERO, Epistemonikos, and MEDLINE) was conducted and revealed that no umbrella reviews on the topic had been published or were in progress. We chose to conduct an overview of systematic qualitative, quantitative, and mixed-studies reviews as this approach provides an overall examination of the existing evidence available for a given topic [13]. We used the JBI methodology and the Preferred Reporting Items for Systematic Reviews and Meta-Analyses (PRISMA) guidelines to structure and report this umbrella review [14, 15]. Although a study protocol had not been previously published for this overview, a priori review protocol was registered in the International Prospective Register of Systematic Reviews (PROSPERO) (number: CRD42023427081). As this is an overview of previously published studies, no ethical approval or patient consent is required.

### Eligibility Criteria

The eligibility criteria were determined based on research questions and were formulated using the population, interventions, comparisons, outcomes, and study design (PICOS) [16], which are presented in Table 1.

**Table 1:** Eligibility criteria

Domains	Inclusion criteria	Exclusion criteria
Population	Health professional students/healthcare students	Not only health professional students and the findings of target population cannot be separately extracted
Intervention	(1)Palliative care education or teaching programs in an academic education context; (2)New palliative care teaching methods in a school education context	(1) education or teaching programs limited to a specific topic/area associated with palliative care, such as advance care planning, pain control, bereavement support, etc.
Comparison	Any alternative approach to teaching about palliative care, usual curriculum content, or no intervention control conditions.	N/A
Outcomes	All measures assessing the effectiveness of palliative care education and learning, such as students’ knowledge, skills, competence, attitude, and qualitative responses to interventions were applicable.	No data reported about the impact of palliative care education and learning on healthcare students
Study design	Systematic qualitative, quantitative, and mixed-studies reviews published in English or Chinese.	Protocols, theses, conference papers

*N/A* not applicable

### Search Strategy and Selection Criteria

An initial search of Google Scholar was made to identify article keywords and the breadth of review studies. Then a search strategy was developed based on Medical Subject Headings terminology and keywords for PubMed (Appendix I), which was later adapted for seven English databases (Web of Science, EMBASE, Cumulative Index to Nursing and Allied Health Literature (CINAHL), Cochrane library, Joanna Briggs Institute (JBI), Education Resources Information Center (ERIC), Epistemonikos) and four Chinese databases (CMB, Wanfang database, CNKI, VIP) in collaboration with an information specialist. Two main strings of terms were developed: one pertained to palliative care education among healthcare students in educational context and its related concepts and keywords, and another string to the methodological filter for reviews and SRs. The initial search was conducted on March 25, 2023. A second search was conducted in Epistemonikos and four Chinese databases on May 19, 2025, and no new literature was included. We conducted a second search only in one English database (Epistemonikos) because it is the largest database of systematic reviews in the health, which maintained by screening multiple information sources, including PubMed, EMBASE, CINAHL, Cochrane, JBI, etc. No language or date restrictions were imposed.

All search results were imported into Covidence software to facilitate de-duplication and screening. Two reviewers (D.J.F and X.L) independently screened the titles and abstracts of the papers for eligibility and then assessed the full text of the remaining records. Discrepancies were resolved through discussion or consulting with a third reviewer (X.Z.S). Moreover, we thoroughly checked the reference lists of the included reviews to identify additional eligible articles. As the aim of the umbrella review was to summarize the current body evidence of palliative care education for healthcare students in undergraduate and postgraduate student programs, all relevant SRs, regardless of primary study overlap, were included. An assessment of the degree of overlap in primary studies was undertaken as per Cochrane guidance [17].

### Quality Assessment of Included Systematic Reviews

The quality of studies was appraised using the JBI Critical Appraisal Checklist for Systematic Reviews and Research Syntheses [15], which consisted of eleven items to determine sources and resources, critical appraisal, methods used, publication bias, and appropriateness of recommendations. Each item was assessed on a categorical scale on compliance: “yes”, “no”, “unsure” and “not applicable” with a potential total positive score of 11 points. Two reviewers (D.J.F and X.L) independently appraised the included SRs and jointly agreed on any discrepancies. The quality of studies was arbitrarily categorized as high (score 9–11), moderate (score 5.5–8.5), or low (score < 5.5).

### Data Extraction

A standardized tool was developed based on recommendations from the JBI and customized for the purposes of this research [15]. The following data were extracted: (1) details of included reviews (e.g. type of SR, databases searched, date range of search, number and types of study included, populations, and methods of synthesis), (2) details about interventions and comparisons, (3) outcomes, including their nature (e.g. qualitative and/or quantitative) and direction (e.g. positive, negative, or no effect). Data extraction was conducted by three reviewers (X.L, D.J.F and X.Z.S). Each paper was extracted independently by two of the three reviewers. Disagreements between the reviewers were resolved through discussion and consensus.

### Data Synthesis

Meta-analysis was not possible because of heterogeneity within and between the included reviews. The narrative synthesis was primarily based on the key findings and conclusions reported in each included review, without reinterpreting individual primary study outcomes. Educational impacts were then mapped to the corresponding Kirkpatrick level to enable consistent comparison across reviews. In order to describe the level of educational impact, we classified the results of each review according to Kirkpatrick’s four-level model [18]. This model proposes four distinct levels as a sequence of ways to evaluate the effectiveness of an educational program: (1) reactions, (2) learning, (3) behavior, and (4) results. Level 1 measures how participants react to the training, such as reactions and satisfaction. Level 2 refers to the extent to which participants change or improve attitudes, knowledge, skills, and/or self-efficacy as a result of attending the interventions. Level 3 (i.e., behavior) is the extent to which students’ learning has been translated into their clinical performance. Level 4 (i.e., results) can be seen as the impact on patient care, organizational or societal change, resulting from the influence of interventions. Two reviewers (D.J.F and X. L) were involved in validating the data synthesis.

### Overlap in Systematic Reviews

The ways of managing overlap across primary studies in SRs depend on the purpose of the overview of SRs and the method of data analysis. Considering the exploratory lens of our overview of SRs and our intent to draw a broad picture of the effects of palliative care education interventions for healthcare students, we simply illustrated the overlap in a citation matrix to visually represent the overlap between primary studies within included SRs. The citation matrix was used to quantify overlap, which was considered to avoid overrepresenting evidence from frequently included primary studies.

## Results

### Study Inclusion

**Fig. 1:**
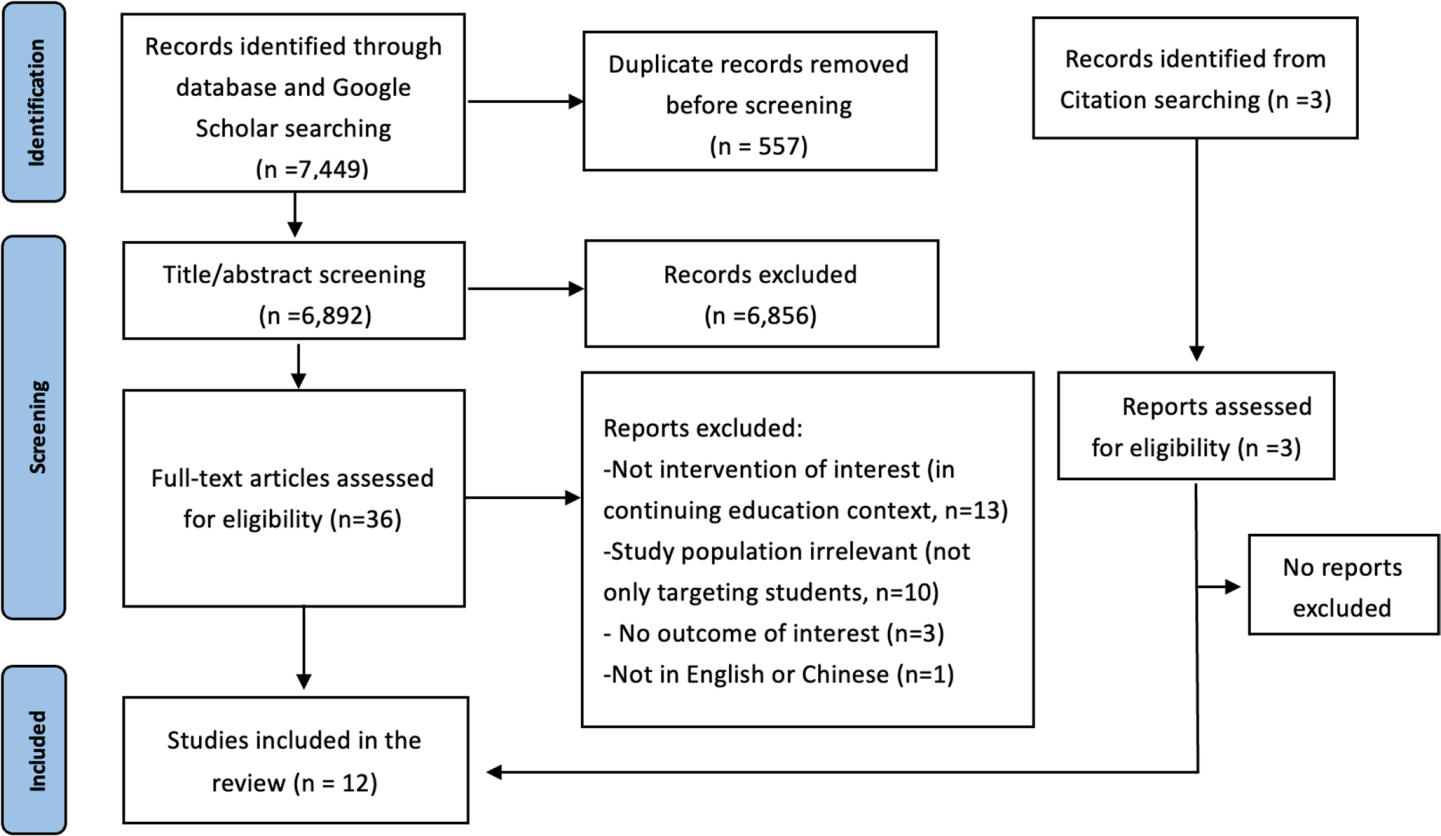
PRISMA 2020 flow diagram for new systematic reviews, including searches of databases and citations

The overall selection of articles is illustrated with the PRISMA flow diagram (Fig. 1). The search identified 7,449 records, of which 6,892 records were retained for eligibility after removal of duplicates. After titles and abstracts screening, 6,856 articles were excluded as they were not meet the predefined eligibility criteria. Thirty-six full-text articles were retrieved for detailed evaluation and 12 were retained for the umbrella review (including three articles found from manually reference checking).

### Characteristics of Included SRs

Of the 12 SRs included in the synthesis, four were described as integrative reviews [11, 12, 19, 20] and eight as SRs by the authors of the included reviews [8–10, 21–25]. Eight of the 12 reviews synthesized findings narratively [11, 12, 19–24]; three combined narrative analysis and meta-analysis [8, 10, 25] and one used meta-synthesis methods [9]. Seven reviews restricted inclusion to English-language studies [10–12, 19–22]; one included study in both English and Chinese [25]; one covered study in English, Swedish, and Finnish [8], and three reviews gave no language restrictions at all [9, 23, 24]. Other basic characteristics of included SRs such as publication year, registration, search information, primary studies included, samples, are summarized in Table 2.

**Table 2:** General characteristics of included systematic reviews

Authors (year)	Review design	Registration	Databases searched; Search period; language	Type and number of studies included	Range years of included studies	Countries/settings	participants/sample size
Hökkä et al., 2022 [8]	systematic review and meta-analysis	NM	CINAHL, ERIC, PubMed, Scopus; From 2009 to 2019; English, Swedish or Finnish	Total: 16 1 RCT, 10 single-group pre-posttest studies, 5 multigroup quasi-experimental studies,	From 2009 to 2019	USA(10), Germany(1), Spain(1), UK(1), Korea (1), Iran (1), Taiwan (1)	1781 undergraduate nursing and medical students.
Donne et al., 2019 [10]	systematic review and meta-analysis	NM	OVID Medline (1946 to 2019), PsycINFO (Ovid 1987-2019), EBM Reviews (Ovid First Quarter 2019), CINAHL Plus (Ebsco 1937-2019), ERIC(ProQuest 1945-2019), EMBASE (Elsevier 1957-2019), and Cochrane through to April 8, 2019. English	Total: 9 8 RCTs, 1 Quasi-RCT	From 1993 to 2017	USA (6), UK (1), Canada (2)	989 undergraduate or postgraduate health professional students
Shaw et al., 2010 [21]	systematic review	NM	MEDLINE, CINAHL, EMBASE; From 1980 to 2008; English	Total: 28 18 single-group pre-posttest studies, 8 multigroup pre- posttest studies, 2 post only	From 1997 to 2009	USA(26), Canada (2)	1216 medical students
Bickel-Swenson 2007 [22]	systematic review	NM	CINAHL, Ovid, MEDLINE, PsycINFO; From 1996 to 2006; English	Total: 9 6 single-group pre-posttest studies, 3 cross-sectional studies	From 1997 to 2005	USA	4826 medical students
Centeno & Rodrı´guez-Nu´n˜ez 2015 [24]	systematic review	NM	Medline, Current Contents Connect, BIOSIS Previews and BIOSIS Citation Index; From 2013 to mid-2015; No language restrictions	Total: 29 4 single-group pre-post design, 2 quasi-experimental multigroup design, 1 post only, 2 cohort studies, 4 cross-sectional, 15 qualitative studies, 1 mixed method study	From 2013 to 2015	USA(7), UK(4), Australia (4), Spain (2), Canada (2), Turkey (2), Germany (2), Korea (2), France (1), Poland (1), Argentina (1), Croatia (1)	4342 medical and nursing students
Boland et al., 2020 [23]	systematic review	PROSPERO No. CRD42018115257	Embase, MEDLINE, PsycINFO, Web of Science, ClinicalTrials.gov, Cochrane, HMIC, ISRCTN registry, BASE, Open Grey, Mednar; From inception to 06 August 2019; No language restrictions	Total: 19 1 RCT, 2 controlled trials, 1 historical control, 14 pre-post design, 1 cross-sectional study	From 2002 to 2018	USA(9), Australia (3), Germany (3), Canada (2), China (2)	3253 medical students
Lippe & Carter, 2015 [19]	integrative review	NM	PubMed, Academic Search Complete, CINAHL, eBook Collection (EBSCOhost), ERIC, MEDLINE, PsycINFO; From 1998 to 2014; English	Total: 14 7 single-group pre-post design (2 mixed-methods studies), 3 quasi-experimental multigroup design, 1 quasi-experimental, longitudinal repeated measures, 2 descriptive studies, 1 qualitative study	From 2001 to 2014	USA (7), Australia (1), Taiwan (1), Canada (1), UK (1), Not reported (3)	1372 medical and nursing students
Ruiz-Pellón et al.,2020 [9]	systematic review and qualitative meta-synthesis	PROSPERO	Medline, Scopus, Web of Science, CINAHL Plus, Dialnet Plus, ERIC and Cuiden Plus; From 2008 to 2018; Language: not reported	Total: 17 2 single-group pre-posttest quantitative studies, 5 mixed-methods studies, 10 qualitative studies	From 2009 to 2018	USA (6), UK (4), Australia (1), Norway (1), South Africa (1), Not reported (4)	606 medical and nursing students
Kukimoto et al.,2023 [11]	integrative review	NM	MEDLINE, CINAHL, ERIC, PsycINFO, and the Cochrane Library’s CENTRAL; From inception to July 2019; English	Total: 15 9 single-group pre-posttest quantitative studies, 1 quantitative controlled study, 1 single-group only posttest study, 1 single group pre-post mixed-methods study, 2 qualitative studies, 1 case review	From 2004 to 2019	USA (8), UK (3), Canada (2), Ireland (1), Swidden and Slovenia (1)	1625 pre-licensure students from any healthcare discipline

*NM* not mentioned, *ERIC* Education Resources Information Center, *CINAHL* Cumulative Index to Nursing and Allied Health, *HMIC* Health Management Information Consortium, *BASE* Bielefeld Academic Search Engine

### Overlap between Included Systematic Reviews

The matrix mapping (Appendix II) included both 12 SRs and their included primary studies included within them. Collectively, 169 primary studies were included in all 12 SRs. The primary studies in the SRs were published between 1993 and 2020. There was a considerable overlap among the primary studies included in the SRs, with 42 studies being included in more than one SR.

### Quality of Included Systematic Reviews

The methodological score of each SR ranged from 6.5 to 10.5. More information can be found in Appendix III. Five reviews evaluated the methodological quality of the primary studies they included, two followed a methodological guideline for SRs. One team published a protocol [9], two registered their reviews prospectively in PROSPERO [9, 23], and three stated compliances with the PRISMA reporting standards [10, 20, 23]. Regarding the search strategy, two teams collaborated with a librarian or information specialist [23, 26]; two reported the detailed syntax for at least one database searched [20, 23] and four checked the reference list of all included articles [9, 10, 22, 23]. In the screening phase, five reviews used two independent reviewers at both the title/abstract and full-text stages [8, 10, 11, 23, 25], and all but one presented a PRISMA-style flow chart of the search results [8–12, 19–21, 23–25].

### Palliative Care Education Interventions and Comparisons

Across the interventions, palliative-care education for healthcare students most often foregrounded core competencies in communication and symptom management. Symptom management content typically addressed pain, breathlessness and nausea. Communication-focused components frequently included delivering bad news, discussing end-of-life preferences, and engaging with families during end-of-life conversations. In addition to these core areas, other covered topics included death and dying, ethical issues, spiritual and psychosocial care, and legal considerations. Some interventions further addressed topics such as grief and bereavement, self-care for healthcare providers, and empathy-building exercises, aiming to cultivate not only clinical proficiency but also emotional resilience and reflective practice (see Appendix IV).

These content areas were delivered through a diverse array of educational interventions aimed at healthcare students, including lecture, seminar, role plays, simulation, online education, pocket card, workshop, videos, clinical rotation, case study, group discussion, and problem-based learning, ect (see Appendix IV). Simulation-based education was the most commonly mentioned teaching methods. According to the clinical rotations of varying length and teaching strategy, Shaw et al. [21] summarized the interventions as low, moderate and high intensity interventions. Low intensity interventions was defined as a short (less than eight hours), single teaching strategy (e.g., workshop, seminar series, educational handout); moderate intensity interventions was defined as longer and/or multifaceted educational strategies, such as simulated patients or a clinical component less than two weeks; high intensity interventions was defined as substantial clinical exposure (greater than two weeks), in addition to multifaceted educational strategies such as rounds, and case discussion. In SR by Boland et al., [23] interventions were summarized in terms of teaching hours as small-scale teaching intervention (with interventions ranging from 1.5 to 10.5 h, with a median of four hours) and large-scale teaching intervention (with interventions ranging from four to five days, with a median of five days).

Most often, no within group or a pre- and post-comparison design were used to explore the effectiveness of the palliative care education intervention. In the included 12 SRs, one review compared the effectiveness of high-fidelity manikin vs. stationary manikins such as standardized persons [20]. One review compared the effectiveness of simulation vs. other teaching methods [25]. The remaining 10 reviews described a combination of different teaching methods for palliative care education and used “no intervention or traditional education” as comparators [8–12, 19, 21–24].

### Effects of Palliative Care Education Interventions

**Fig. 2:**
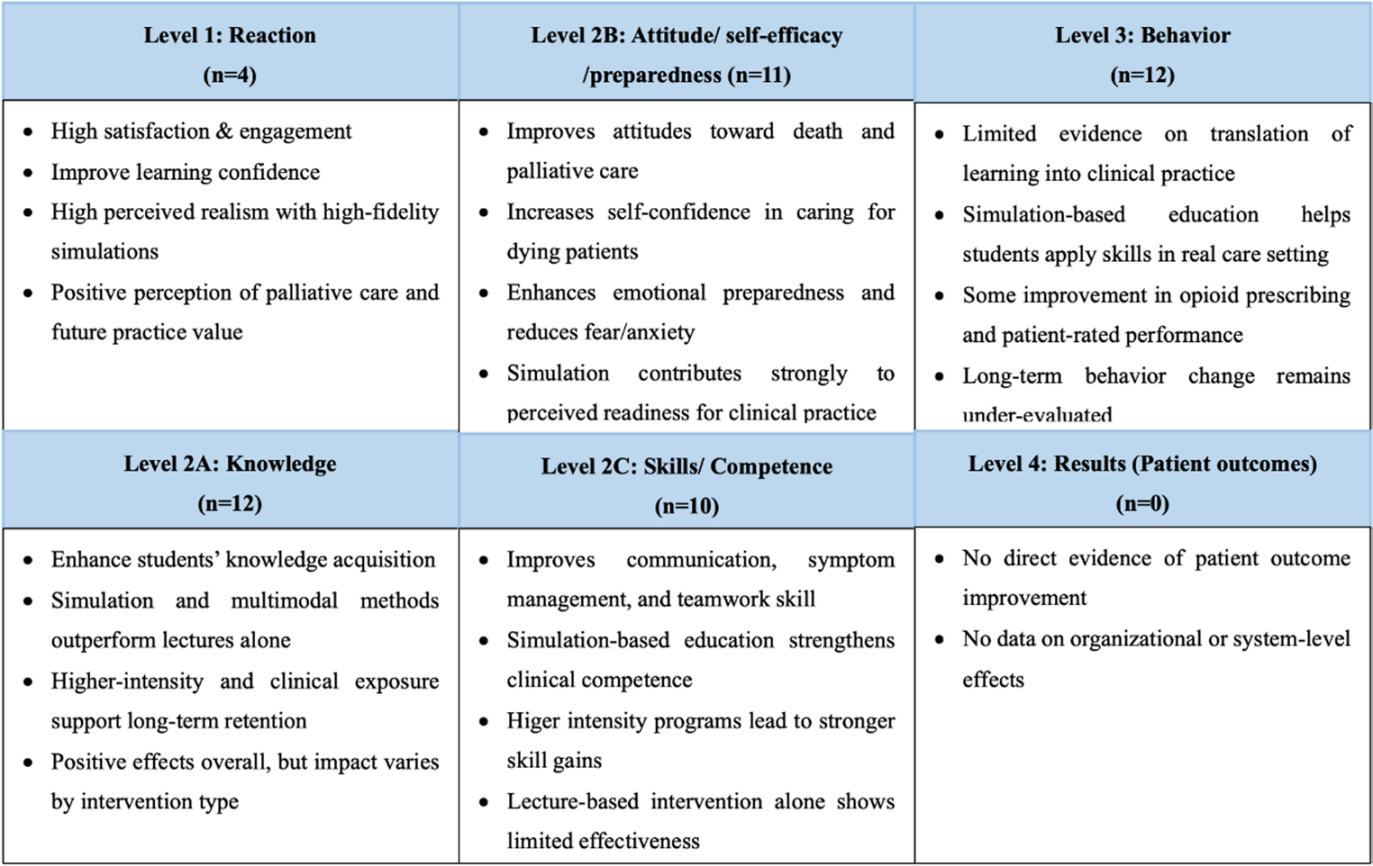
Summary of outcomes of palliative care education mapped to Kirkpatrick’s Four-Level Training Evaluation Model

All included SRs were classified using Kirkpatrick’s four-level model (Table 3). Four reviews examined students’ reactions to the education training (Kirkpatrick 1) [9, 11, 20, 24]. All 12 reviews examined students’ learning outcomes (Kirkpatrick 2) such as knowledge, attitude, skills [8–12, 19–25]. Five reviews examined learner behavioral change and performance in practice (Kirkpatrick 3) [8, 10, 21, 23, 24] and none of the SRs examined changes in patient care and organizational change (Kirkpatrick 4). Overall, positive outcomes are more prevalent than negative outcomes or those showing no significant effect. Figure 2 illustrates the mapping of the results to Kirkpatrick’s four-level model. The detailed findings of each SR are presented in Appendix V.

**Table 3:** Interventions, comparisons and level of evaluation/outcomes

Authors (year)	Interventions	Comparisons	Levels of evaluation according to Kirkpatrick Model
Hökkä et al., 2022 [8]	Any intervention of teaching or learning methods in palliative care that combine guided teaching within classroom or clinical learning (such as contact teaching and films, didactic lectures, bedside patient care and interactive discussions, using lectures, interactive discussions, patient exposure and role-play)	At least one control group or pre- and post-test measurement. The group has a standard education or no intervention control conditions.	(1) attitudes and self-efficacy (Kirkpatrick 2a) (2) knowledge (Kirkpatrick 2b) (3) competencies (Kirkpatrick 2b) (4) behavior (Kirkpatrick 3)
Donne et al., 2019 [10]	A number of intervention types including a lecture, seminar, role plays, online education, computer-based decision aid, pocket card, workshop, follow-up phone call from a facilitator, group discussion, teamwork, simulation, and patient exposure.	Usual curriculum content, or no intervention control conditions.	(1) attitudes and confidence (Kirkpatrick 2a) (2) knowledge (Kirkpatrick 2b) (3) skills (Kirkpatrick 2b) (4) behavior (Knowledge into practice) (Kirkpatrick 3)
Shaw et al., 2010 [21]	Any type of end-of-life care curriculum, including didactic sessions, simulated patients or role play, clinical rotations of varying length (1 week to 1 year longitudinal) combined with didactic teaching, seminar series or Web modules, workshops or retreats and a brief 10-minute intervention and pocket card.	A standard education or no intervention control conditions.	(1) attitudes, confidence, self-perceived competence and comfort (Kirkpatrick 2a) (2) knowledge (Kirkpatrick 2b) (3) skills (Kirkpatrick 2b) (4) behavior (Kirkpatrick 3)
Bickel-Swenson 2007 [22]	End-of-life teaching including didactic sessions, case studies, small group discussions, video learning	Pre- and post-test measurement	(1) confidence, Preparedness (Kirkpatrick 2a) (2) knowledge (Kirkpatrick 2b) (3) competency (Kirkpatrick 2b) (4)skills (Kirkpatrick 2b)
Boland et al., 2020 [23]	Any type of education including eLearning lecture, videos, clinical rotation, workshops, simulation, role play, small group teaching, bedside teaching, reflective essays	Any comparators were considered for inclusion. Likely to be no, different or less education	(1) knowledge (Kirkpatrick 2b) (2) behavior (Kirkpatrick 3)
Centeno and Rodrı´guez-Nu´n˜ez 2015 [24]	A variety of educational interventions on palliative care or end-of-life care such as lecture, simulation, film, clinical rotation.	Pre- and post-test measurement or no intervention control conditions.	(1) reactions after learning experiences (Kirkpatrick 1) (2) comfort, preparedness, confidence, attitude (Kirkpatrick 2a) (3) knowledge (Kirkpatrick 2b) (4) skills (Kirkpatrick 2b) (5) behavior (Long-term influence of palliative care teaching on current clinical practice) (Kirkpatrick 3)
Lippe and Carter, 2015 [19]	The implementation of teaching strategies, as well as outcome measurements of students’ learning based on implementation of those strategies.	Pre- and post-test measurement or no intervention control conditions.	(1) attitude, self-confidence or self-efficacy, awareness/appreciation (Kirkpatrick 2a) knowledge (Kirkpatrick 2b).
Ruiz-Pellón et al.,2020 [9]	A variety of palliative and end of life care pedagogical activities such as lecture, simulation	classroom-based teaching or no intervention control conditions.	(1) reactions (Kirkpatrick 1) (2) attitude (Kirkpatrick 2a) (3) knowledge (Kirkpatrick 2b) competencies acquired on death and end-of-life (Kirkpatrick 2b).
Kukimoto et al.,2023 [11]	A variety of palliative and end of life care teaching strategies such as simulation, role-play, workshop, clinical rotation, case study and training)	pre- and post-test measurement	(1) reactions (Kirkpatrick 1) (2) attitudes, perceptions and confidence (Kirkpatrick 2a) (3) knowledge (Kirkpatrick 2b) competencies and skills (Kirkpatrick 2b).
Kirkpatrick et al.,2017 [20]	High-fidelity Manikins	Stationary manikins, (Standardized persons)	(1) reactions: satisfaction with the instructional method (Kirkpatrick 1) (2) confidence (Kirkpatrick 2a) (3) knowledge (Kirkpatrick 2b) skills (Kirkpatrick 2b).
Lippe et al., 2018 [12]	Any educational interventions (workshops, programs, curricula, simulations, patient care experiences, dedicated courses) specifically targeted to address topics related to palliative, EOL, or hospice care	pre- and post-test measurement	(1) attitude, self-efficacy/confidence/perceived competence, preparedness (Kirkpatrick 2a) (2) knowledge (Kirkpatrick 2b) (3) skills, competence (Kirkpatrick 2b)
Wang Yan,2022 [25]	Simulation, role play	Other teaching methods such as lectures, case study, or pre- and post-test measurement	(1) attitudes (Kirkpatrick 2a) (2) knowledge (Kirkpatrick 2b) communication skills (Kirkpatrick 2b)

#### Level 1: Reactions with Palliative Care Education Interventions

As shown in Fig. 2, four SRs described positive healthcare students’ reactions with palliative care education interventions, mostly in terms of student satisfaction with simulation experience of palliative care. Healthcare students experienced high strong engagement and emotional resonance during training [20], and perceived improved readiness for providing palliative care in future practice [9, 11, 24]. Higher-fidelity simulations—particularly those using standardized patients or real actors—were perceived as more realistic and educationally valuable than static manikins, especially in recognizing key patient symptoms such as pain and dyspnea [20]. The training was also seen as meaningful, confidence-enhancing and supportive of professional identity development [9, 24]. Additionally, hospice-based interprofessional practice placement increased students’ understanding of both the role of their profession and those of other professions [11].

#### Level 2: Learning

Across the included SRs, all the educational outcomes mapped to Kirkpatrick Level 2 (Learning) and demonstrated improvement in students’ knowledge, attitude/self-efficacy/preparedness, and skills/competence, particularly when simulation or multimodal teaching approaches were used (Fig. 2).

### Knowledge

All included SRs reported improvements in participants’ ability of knowledge acquisition following palliative care education. The included SRs reported that a variety of teaching or learning interventions including dedicated courses, simulations, eLearning, patient care experiences, interprofessional education, problem-based learning [8, 10–12, 22]. Meta-analyses demonstrated significant gains, particularly favoring simulation-based approaches over traditional didactic methods [10, 25]. Narrative analysis likewise indicated consistent and positive outcomes regarding knowledge enhancement following students’ engagement in palliative care education interventions, although several reviews noted heterogeneity in effect sizes across primary studies [11, 12, 19, 21, 23]. Moreover, higher-intensity and clinical immersion experiences supported broader and more sustained knowledge retention compared with low-intensity or standalone lecture-based teaching, including mandatory clinical modules outperforming purely didactic formats [21, 23].

### Attitude/self-efficacy/preparedness

All but one included SRs reported participants’ changes in attitude/perceptions/self-efficacy/preparedness (Fig. 2). Two meta-analysis demonstrated significant improvement in attitudes toward death and caring for dying a person, particular when diverse or simulation-enhanced teaching strategies were applied [8, 10]. Narrative syntheses across reviews further supported positive shifts in attitudes toward palliative care and end-of-life involvement, albeit with variability across interventions and study designs [8, 10–12, 19]. Notably, two SRs demonstrated non-significant changes [12, 24], and one SR demonstrated that students had both positive and negative attitudes towards participation in end-of-life programs [9]. Seven SRs reported Student’s self-confidence were reported in seven SRs, mainly related to end-of-life care curricula, palliative care curricula, simulation, pocket card, small group activities, inpatient hospice care [8, 10–12, 19–21]. Among them, one SR including two studies reported an increase in student’s self-confidence for providing end-of-life care to patients [19], though only one study found significance (*P* = 0.05). Six reviews indicated students’ emotional preparedness for clinical eventualities improved, including reduced fear and avoidance of dying patients and more comfort with symptom management discussions [1–21, 24]. However, 39% of students still expressed feeling unprepared to manage complex psychosocial and family concerns related to dying [22].

### Skills/competence

All but two included SRs reported improvement in participants’ skills or competence acquisition (Fig. 2). Positives outcomes were demonstrated across a range of skills, including communication, pain management, teamwork, and addressing psychosocial concerns [12, 22, 24]. Palliative care courses and diverse educational approaches—including simulations, role-play, interprofessional education, problem-based learning, clinical rotations, and computer-based decision aids—consistently led to significant improvements in students’ skills and clinical competence [8–12, 20–22, 24]. Simulation-based education was the most commonly mentioned teaching methods which significantly improved students’ communication skills [20, 21, 24], students’ competencies acquired on death and end-of-life care [9, 25], and increased students’ quality performance [8]. Furthermore, moderate intensity interventions (multifaceted educational strategies, such as simulated patients or a clinical component, more than eight hours and less than two weeks) with simulated patients showed objective improvements in end-of-life communication skills [21]. Courses in basic science and patient interviewing, as well as problem-based learning seminars, were least effective in affecting competency in end-of-life care [22].

#### Level 3: Behavior

Evidence for behavioral change was limited, with only five reviews reporting outcomes at this level [8, 10, 21, 23, 24] (Fig. 2). Simulation-based education appeared to facilitate the transfer of communication and clinical skills into real clinical encounters [10]. Educational modules on pain and symptom management produced improvements in opioid prescribing at 6 months when assessed by chart audit [21]. Evidence also suggested improvements in patient-rated performance during advance directive discussions when decision-aid tools were used in training [8, 23]. One review documented a narrative summary of one qualitative study exploring the long-term influence of palliative care teaching on current clinical practice and showed that undergraduate students attending a palliative care clinical elective make a difference in treating dying patients after 3.5 years when they worked as junior doctors [24].

#### Level 4: Results (Patient outcomes)

No study analyzed whether the act of palliative care education interventions had an effective and efficient impact on patient care and organizational or societal change.

## Discussions

### Main Findings

Our review systematically synthesised evidence from 12 systematic reviews, including qualitative, quantitative, and mixed-method studies, incorporating findings from 165 primary studies to examine the impact of palliative care education on healthcare professional students. Utilizing Kirkpatrick’s Four-Level Training Evaluation Model as a standardized and validated framework, we sought to enhance our comprehension of the effect of palliative care training in undergraduate and postgraduate programs among healthcare professional students. Given the variability within and between the included reviews, we employed a narrative synthesis to present the results. The most frequently emphasized areas in palliative care intervention were symptom management and communication skills. Across systematic reviews, the collective outcomes consistently indicated a positive effect of palliative care education interventions on healthcare professional students’ attitudes, knowledge, skills, and/or self-efficacy. However, few studies have explored how the education interventions affected the students’ behavior and patients’ outcomes.

Although most palliative care education programs emphasize symptom management and communication skills, these content areas may not be sufficient to meet the complex and multifaceted needs of real-world palliative care practice. Palliative care is a patient-centred approach aimed at relieving suffering and enhancing quality of life through comprehensive physical, psychological, social, and spiritual support [1]. However, many existing education programs provide limited attention to topics such as ethical and legal issues, psychosocial and spiritual concerns, bereavement, and self-support. Addressing these dimensions is essential for preparing healthcare students to deliver holistic and person-centred care, as well as to develop the emotional resilience and ethical sensitivity required in palliative care settings [5, 27]. To better prepare future healthcare professionals, palliative care education should integrate these often-overlooked elements alongside clinical and communicative skills, thereby supporting a more comprehensive and reflective approach to end-of-life care.

This review found that the reported outcomes of palliative care education interventions in the included SRs predominantly fell within level 2 of Kirkpatrick’s model: the extent to which participants alter or improve their attitudes, knowledge, skills, and/or self-efficacy. Notably, the primary outcomes of concern are knowledge and skill acquisition. Integrating various teaching methods significantly enhanced students’ knowledge/skill acquisition. The advantages of combinatorial teaching methods, which increase participants’ interest in learning, and often increase simulation education, enable participants to effectively acquire knowledge. The use of simulation allows participants to practice and apply their knowledge and skills [28]. A key element in the simulation is debriefing which allows students to analyze the dynamics of the situation, thus making them more familiar with the knowledge and improving their skills [29, 30]. At this level of evaluation, simulation-based teaching is the most common approach. Compared to traditional educational models, simulation-based teaching can bridge the gap between clinical practice and theoretical content [31]. Existing evidence demonstrated that simulation-based palliative care educational interventions have had significant positive effects on students’ knowledge, communication skills, and alleviation of fear when interacting with terminally ill patients [32–34]. Some evidences suggest that simulation-based teaching offers an effective and acceptable teaching environment that allows participants to “firsthand experience” in care of dying patients and their families [35, 36]. Notably, this immersive experience proves particularly beneficial in end-of-life communication training. This is because it allows for the simulation of realistic conversations infused with body language and emotions, which are challenging in traditional educational methods [37]. Moreover, the teaching environment provided by simulation-based learning is safe, mitigating risks that the participants face related to psychological safety [38]. Psychological safety means that participants feel free to take risks without worrying about the negative outcomes [38], which can enhance students’ interest and participation in the simulation-based learning process [39, 40].

Limited evidence was available regarding the impact of palliative care education on students’ behavior acquisition (level 3), and none looked at the impact of the interventions from patients’ outcomes and organizational perspectives. This may be because measuring the clinical impact of teaching interventions requires rigorous long-term follow-up, including tracking participant behavior and outcomes over time, which would increase both the time and cost of the study [41, 42]. However, given the growing need for palliative care, it is crucial to ascertain whether palliative care education interventions actually improve clinical practice and patient outcomes. While changes in knowledge, attitudes and skills at level 2 are important, assessments at level 3 and level 4 could offer a more comprehensive understanding of the true benefits of palliative care educational interventions [43, 44]. In addition, by assessing level 3 and level 4 outcomes, organizations can identify the long-term facilitators and barriers to palliative care education interventions, thereby fostering sustained improvements in the quality of care provided by the organization [45]. Future research should not only focus on the cost, time and overall requirements of palliative care educational interventions but also on the practicability of educational interventions, that is, whether students’ behaviors can be translated into clinical manifestations and improve patient outcomes. At the same time, our findings indicate that current palliative care education is largely effective in fostering foundational learning outcomes, yet greater emphasis on higher-level outcomes is needed to inform curriculum design that meaningfully supports the transfer of learning into clinical practice.

### Implications for Palliative Care Education and Future Research

With an ageing population and higher rates of non-communicable diseases, there is an increased need for palliative care. All healthcare professional students are expected to possess core competencies in palliative care. However, current medical curricula are overcrowded, and education and training in the physiological, psychological, and psychosocial aspects of dying have been grossly inadequate in most medical schools globally. Potential barriers include lack of teaching time and difficulties in providing clinical placements [42]. Another major barrier factor is few and weak policies advocating for the inclusion of palliative care education in medical curricula [6].

Across our review, there are some strategies and methods available to improve the coverage of palliative care education programs among healthcare professional students. First, palliative care education content has been included in mandatory courses or integrated into core courses in some countries and is recommended to be further strengthened and more widely implemented as palliative and end-of-life care evolves into a mature discipline. Such integration ensures that palliative care becomes an essential component of healthcare training. Second, curriculum planners should embed palliative care learning longitudinally across stages of training, ensuring foundational exposure in pre-clerkship years and progressive development of clinical communication and team-based competencies during clerkship and postgraduate practice. While existing international framework and curricular initiatives have outlined core components of palliative care education, a standardized global palliative care curriculum should be developed and made freely available so that all countries can use this resource for basic training in palliative care principles and build on it to adapt to local needs and circumstances. For countries without local palliative care expertise, external technical assistance can play a key role in establishing basic training programs and building local capacity. Third, interdisciplinarity approaches have increasingly been integrated into palliative care education and are considered an essential aspect for enhancing healthcare professional students’ capacity in palliative care. Future curricula would encourage students to appreciate the roles of different professionals and promote the teamwork needed to provide palliative care. Finally, simulation-based teaching, a common strategy identified in this review, may be scaled in diverse contexts by adopting cost-efficient formats such as standardized patients and virtual simulation. Meanwhile, the limited evidence at Kirkpatrick levels 3 and 4 underscores the need for longitudinal, workplace-based and mixed-methods evaluations that examine the transfer of learning into clinical behavior and, ultimately, patient outcome.

### Strengths and Limitations

Our overview identified several strengths. Firstly, the comprehensive nature of our review encompassed a wide range of palliative care education programs, including those published in Chinese or English. Secondly, we employed a rigorous systematic literature selection process, independently conducted by two reviewers (D.J.F and X. L) to minimize subjective bias. Thirdly, we used the JBI Critical Appraisal Checklist for Systematic Reviews and Research Syntheses to evaluate the methodological quality of the included studies, ensuring a robust assessment.

However, there were also some limitations. Many of the included studies were based on low-quality evidence, limiting their reliability. Additionally, we opted for narrative synthesis rather than meta-analysis due to the significant heterogeneity within and between the included studies. This approach may have introduced bias into our findings, limiting their generalizability and interpretability. In addition, primary study overlap across reviews may have resulted in overrepresentation of certain findings. Finally, the absence of a comparison intervention in most studies hindered our ability to determine the effectiveness of palliative care education interventions. These limitations should be considered when applying our findings to curriculum planning and institutional decision-making.

## Conclusion

Palliative care education programs had positive effects on healthcare professional students’ knowledge, attitudes, self-efficacy, skills, and competence. However, few studies have explored the true impact of palliative care educational interventions, namely Kirkpatrick level 3 (behavioral change) and level 4 (patients’ outcome or impact on organization). We recommend further research to identify facilitators and barriers to palliative care education interventions for healthcare professional students and to determine the long-term effects and mechanisms of palliative care education intervention, including longitudinal and organization-based evaluations that assess the transfer of learning into clinical practice and patient outcomes.

## Supplementary Information

Below is the link to the electronic supplementary material.

Supplementary file 1 (XLSX 96.0 KB)

## Data Availability

Research data are not shared.
